# Mitochondrial DNA Fragmentation and Risk of Non-Hodgkin Lymphoma

**DOI:** 10.1001/jamanetworkopen.2023.26885

**Published:** 2023-08-02

**Authors:** H. Dean Hosgood, Meghan Davitt, Richard Cawthon, Stephanie J. Weinstein, Batel Blechter, Jason Y.Y. Wong, Mohammad L. Rahman, Wei Hu, Satu Männistö, Demetrius Albanes, Nathaniel Rothman, Qing Lan

**Affiliations:** 1 Department of Epidemiology and Population Health, Albert Einstein College of Medicine, Bronx, New York; 2 Department of Human Genetics, University of Utah, Salt Lake City; 3 Division of Cancer Epidemiology and Genetics, National Cancer Institute, National Institutes of Health, US Department of Health and Human Services, Bethesda, Maryland; 4 Department of Public Health Promotion, National Institute for Health and Welfare, Helsinki, Finland

## Abstract

**Importance:**

Research suggests that increased mitochondrial DNA copy number (mtDNAcn) is associated with increased risk of non-Hodgkin lymphoma (NHL); however, no studies to date have evaluated whether the mitochondrial DNA fraction with breaks (mtDNAfb) is associated with risk of NHL.

**Objective:**

To evaluate the association of mtDNAfb with NHL risk.

**Design, Setting, and Participants:**

This nested case-control study, which used prospectively collected samples as part of baseline enrollment (from 1985 through 1988) of 29 133 men who smoked for the Alpha-Tocopherol, Beta-Carotene Cancer Prevention (ATBC) Study conducted in southwest Finland, included 107 incident NHL cases and 107 controls (matched on date of birth ±5 years). Analyses were conducted from January to September 2022.

**Exposure:**

High-throughput real-time polymerase chain reaction assays quantifying mtDNAfb.

**Main Outcomes and Measures:**

Incident NHL cases were identified in the ATBC Study through April 30, 2002, using the Finnish Cancer Registry and the Register of Causes of Death. The mtDNAfb was quantified and categorized based on the median, tertile, and quartile distributions among controls. Odds ratios (ORs) and 95% CIs were estimated using conditional logistic regression models to assess the associations between categorized mtDNAfb and future risk of NHL, controlling for age, body mass index, number of cigarettes smoked per day, number of pack-years, and mtDNAcn.

**Results:**

A total of 29 133 men (median [IQR] age, 57.2 [52.6-62.5] years) participated in ATBC Study. Higher mtDNAfb was associated with an increased risk of NHL (median OR, 2.89; 95% CI, 1.40-5.93) in a dose-dependent manner (quartile 2 vs 1 OR, 1.24; 95% CI, 0.43-3.40; quartile 3 vs 1 OR, 3.58; 95% CI, 1.39-9.24; quartile 4 vs 1 OR, 3.42; 95% CI, 1.30- 8.99; *P* = .004 for trend).

**Conclusions and Relevance:**

This study’s findings suggest that increased mtDNAfb is associated with an increased future risk of NHL. Additional studies are needed to confirm these findings, particularly among women and nonsmokers.

## Key Points

**Question:**

Is the mitochondrial DNA fraction with breaks (mtDNAfb) associated with non-Hodgkin lymphoma (NHL) risk?

**Findings:**

In this nested case-control study of 107 incident NHL cases and 107 controls within the prospective Alpha-Tocopherol, Beta-Carotene Cancer Prevention Study (consisting of 29 133 men), a higher mtDNAfb was associated with a 2.9 times increased risk of NHL with a dose-response association between quartiles of mtDNAfb and risk of NHL.

**Meaning:**

These findings suggest that an increased mtDNAfb may be associated with an increased risk of NHL.

## Teaser

This case-control study evaluates whether the fraction of mitochondrial DNA copies with breaks is associated with risk of non-Hodgkin lymphoma in a population of men from Finland.

## Introduction

The incidence of non-Hodgkin lymphoma (NHL) has been increasing in recent decades in all industrialized countries.^1^ Although risk factors such as certain autoimmune conditions and occupational exposures such as benzene, trichloroethylene, and formaldehyde are known to increase the risk of NHL, the etiology of this disease is still not well characterized.^2,3,4,5,6^ Previous research^7^ has demonstrated an association between increased mitochondrial DNA copy number (mtDNAcn) and increased risk of NHL. In healthy, nondamaged cells, mitochondria, eukaryotic organelles in the body involved in energy production through the production of adenosine triphosphate, have 2 to 10 copies of their genomes, mtDNA.^8^ Mitochondrial DNA differs from genomic DNA in that it lacks introns and protective histones, leaving it more vulnerable to oxidative damage from reactive oxygen species or other environmental stressors.^8,9^ The mtDNAcn increases when mitochondria sense damage, because mitochondria have limited DNA repair capacity with an increased number of mutations in response to damage.^10,11^ When exposed to environmental stress, mitochondria shift toward fragmentation (fission) as a prosurvival mechanism, with studies showing linear associations between fragmentation and cytotoxic effects when exposed to cell stress.^12^ This study sought to evaluate whether mtDNA fraction with breaks (mtDNAfb) is associated with the risk of developing NHL using prediagnostic blood samples collected prospectively in the Alpha-Tocopherol, Beta-Carotene Cancer Prevention (ATBC) Study.

## Methods

### Study Population

The ATBC Study, a cancer prevention trial conducted by the US National Cancer Institute (NCI) and the National Public Health Institute of Finland, has been previously described.^13^ The study was approved by institutional review boards at the NCI and the National Public Health Institute of Finland. All participants provided written informed consent. This case-control follows the Strengthening the Reporting of Observational Studies in Epidemiology (STROBE) reporting guideline for observational studies.

Briefly, the participants were randomized to receive 1 capsule daily (for 5-8 years) containing 1 of the following: 50 mg of vitamin E, 20 mg of beta carotene, both 50 mg of vitamin E and 20 mg of beta carotene, or a placebo (ie, an inactive capsule that appeared identical to the vitamin capsules). A total of 29 133 male smokers aged 50 to 69 years were recruited from 1985 through 1988 from southwest Finland. Using the Finnish Cancer Registry and the Register of Causes of Death, which provide nearly 100% of cancer case ascertainment in Finland,^14^ incident NHL cases (n = 107) were identified in the ATBC Study through April 30, 2002. Controls (n = 107) were randomly selected from the ATBC Study members who were alive and free of cancer at the time of the case diagnosis and individually matched to cases on date of birth (±5 years). DNA was extracted from whole blood using the phenol-chloroform method,^15^ and a fluorescence-based quantitative polymerase chain reaction (qPCR) determined mtDNAcn,^16^ whereas mtDNAfb was determined by a high-throughput qPCR assay similar to those described by others quantifying human mtDNA deletion frequency.^17,18^ The primers for qPCR measurement of mtDNAfb were mt10041u: GTACCGTTAACTTCCAATTAACTAGTTTTGGCA-3′ and mt10047d: 5′-TTGATTATTAAAATTAAGGCGAAGTTTATTACTCTTTTCTG-3′, each present at a final concentration of 250 nM. Additional components of the qPCR were 1 × Titanium Taq DNA Polymerase (Takara Bio Inc), 1 × Klentaq1 Reaction Buffer pH 7.9 (DNA Polymerase Technology), 200 μM of each dNTP, 1 M of betaine, 100 ng/μL of bovine serum albumin (New England Biolabs), and 1 × SYBR Green I (Thermo Fisher Scientific). The 3.5 mM of magnesium chloride provided by the Klentaq1 buffer was supplemented with magnesium chloride to a final concentration of 5 mM. For each DNA sample, 3 replicate reactions were preincubated with *Taq*I restriction endonuclease (New England Biolabs), 0.11 units per 10-μL reaction, for 10 minutes at 65 °C before the initiation of thermal cycling; another 3 replicate reactions were subjected to the same protocol but without the restriction digestion step. Approximately 0.1 ng of total cellular DNA from each participant was assayed per reaction well. Standard curve total cellular DNA (reference DNA) amounts per 10-μL final reaction volume were 1, 0.333, 0.111, 0.037, and 0.0123 ng. All reference DNA used for relative quantification of the experimental samples’ amplification signals were restriction nuclease digested before thermal cycling. Thermal cycling and amplification signal acquisition were performed on a Bio-Rad CFX384 real-time PCR detection system. In this relative quantification approach, each experimental DNA sample’s mtDNA total signal was expressed as the number of nanograms of the reference DNA that matched the restriction-digested experimental sample for copy number of the PCR target template, and the experimental sample’s mtDNA copies with breaks signal was expressed as the number of nanograms of the reference DNA that matched the undigested experimental sample for copy number of the PCR target template. The latter signal divided by the former signal is the fraction of mtDNA copies with breaks for that experimental DNA sample. Thermal cycling was as follows: 65 °C for 10 minutes, 94 °C for 3 minutes, 92 °C for 15 seconds, 60 °C 30 for seconds, and 70 °C for 20 seconds with signal acquisition, with these steps repeated for a total of 30 cycles. Cases and their matched controls were blindly assayed consecutively within the same batch. The mtDNAcn data were available for 97 case-control pairs, whereas mtDNAfb information was available for 95 case-control pairs.

### Statistical Analysis

Spearman correlation was used to evaluate the association between mtDNAfb with body mass index (BMI; calculated as weight in kilograms divided by height in meters squared), number of cigarettes smoked per day, pack-years of smoking, and mtDNAcn. The mtDNAfb, BMI, number of cigarettes smoked per day, pack-years of smoking, and treatment group (alpha tocopherol vs beta carotene) were compared by case-control status using χ^2^ and 2-sided *t* tests when normality was established through visual inspection and by Mann-Whitney *U* testing when the normality assumption was not met. mtDNAfbs were categorized by median, tertiles, and quartiles based on the distribution among controls. Odds ratios (ORs) and 95% CIs were estimated using conditional logistic regression models to assess the associations between future risk of NHL and categorized mtDNAfb with an a priori level of significance of a 2-sided *P* < .05. The mtDNAfb tertile indicator variables were included in conditional logistic regression models to test for ordinal trend. Models were adjusted for age, pack-years of smoking, number of cigarettes smoked per day, BMI, and mtDNAcn. Similar analyses were performed to evaluate the association between mtDNAcn and NHL. To evaluate for the potential effects of the vitamin supplementation that initially took place in the trial, stratified analyses by supplementation arms were performed. Exploratory analyses were conducted excluding cases (and their matched controls) diagnosed within the first year and first 2 years of follow-up after blood sample collection. Finally, we tested for a multiplicative interaction between median mtDNAfb and median mtDNAcn and risk of NHL by including both the independent effects of mtDNAcn and mtDNAfb and the interaction term in the conditional logistic regression models. All analyses were conducted using Stata software, version 17.0 (StataCorp LLC) from January to September 2022.

## Results

The 29 133 men who participated in the ATBC Study had a median (IQR) age of 57.2 (52.6-62.5) years, smoked a median (IQR) of 20.4 (13-25) cigarettes daily, and had smoked for a median (IQR) of 35.9 (30-43) years.^13^ A total of 107 incident NHL cases were identified during the follow-up time. Cases and controls were similar with respect to BMI; smoking habits, including number of cigarettes per day and pack years of smoking; and ATBC Study randomized treatment group (Table 1). Cases had increased median (IQR) mtDNAcn compared with controls (0.99 [0.87-1.31] vs 0.91 [0.83-1.02]; *P* < .001). Median (IQR) mtDNAfbs were also increased among cases compared with controls (0.21 [0.18-0.28] vs 0.18 [0.15-0.24]; *P* < .001). mtDNAfb was not associated with BMI (cases: ρ = 0.15, *P* = .14; controls: ρ = −0.13, *P* = .22), number of cigarettes smoked per day (cases: ρ = −0.10, *P* = .31; controls: ρ = −0.02, *P* = .81), or total pack-years (cases: ρ = −0.09, *P* = .37; controls: ρ = 0.05, *P* = .63) among controls or cases (eTable 1 in Supplement 1). There was a weak association between mtDNAcn and mtDNAfb among controls (ρ = 0.08; *P* = .006) but not in cases (ρ = 0.0025; *P* = .63).

**Table 1.** Characteristics of Non-Hodgkin Lymphoma Cases and Controls From the Alpha-Tocopherol, Beta-Carotene Cancer Prevention Study

Demographic characteristic	Controls (n = 97)	Cases (n = 97)	*P* value
BMI, mean (SD)	26.1 (3.5)	26.4 (4.2)	.80
Smoking status, median (IQR)			
No. of cigarettes smoked per day	20 (15-25)	20 (15-25)	.84
Pack-years of smoking	36 (26-46)	37 (23-50)	.87
Supplementation group, No. (%)			
Placebo	26 (27.4)	26 (27.4)	.94
Alpha tocopherol alone	22 (23.2)	25 (26.3)	
Beta carotene alone	25 (6.3)	22 (23.2)	
Alpha tocopherol and beta carotene	22 (23.2)	22 (23.2)	
Mitochondrial DNA copy number, median (IQR)	0.9075 (0.8325-1.0150)	0.9895 (0.8725-1.3100)	<.001
Mitochondrial DNA fraction with breaks, median (IQR)	0.183 (0.150-0.235)	0.213 (0.179-0.275)	<.001

Abbreviation: BMI, body mass index (calculated as weight in kilograms divided by height in meters squared).

An increase in mtDNAfb was associated with an increase in the risk of developing NHL (Table 2). When categorizing mtDNAfb based on the median among controls, participants with a higher mtDNAfb had a 2.9-fold increased risk of developing NHL compared with participants with a lower mtDNAfb (median OR, 2.89; 95% CI, 1.40-5.93). Similarly, participants in the highest 2 tertiles of mtDNAfb had an increased future risk of NHL (tertile 2 vs 1 OR, 1.78; 95% CI, 0.80-3.99; tertile 3 vs 1 OR, 2.08; 95% CI, 0.95-4.53) compared with those in the lowest tertile (Table 2). When categorizing mtDNAfb based on quartiles among controls, participants in the highest 3 quartiles experienced an increased risk of developing NHL compared with participants in the lowest quartile, with an observed dose-response association (quartile 2 vs 1 OR, 1.24; 95% CI, 0.43-3.40; quartile 3 vs 1 OR, 3.58; 95% CI, 1.39-9.24; quartile 4 vs 1 OR, 3.42; 95% CI, 1.30-8.99; *P* = .004 for trend). Similar associations between mtDNAfb and NHL future risk were observed after adjusting for treatment group (eTable 2 in Supplement 1), as well as when exploratory analyses excluded those diagnosed within 1 year of blood draw (eTable 3 in Supplement 1) and 2 years of blood draw (eTable 4 in Supplement 1).

**Table 2.** Risk of Non-Hodgkin Lymphoma Associated With mtDNAfb

mtDNAfb	No. of controls	No. of cases	Conditional logistic regression		Conditional logistic regression adjusted for pack-years of smoking, number of cigarettes per day, and BMI		Conditional logistic regression adjusted for mtDNA copy number, pack-years of smoking, number of cigarettes per day, and BMI	
			OR (95% CI)	*P* value	OR (95% CI)	*P* value	OR (95% CI)	*P* value
Median analysis^a^								
Less than median (0.116-0.2044)	47	25	1 [Reference]	NA	1.00 [Reference]	NA	1 [Reference]	NA
Median or greater (0.2045-0.557)	48	70	3.20 (1.57-6.51)	.001	2.89 (1.40-5.93)	.002	4.03 (1.74-9.33)	.001
Tertile analysis^a^								
Tertile 1 (0.116-0.168)	30	18	1 [Reference]	NA	1.00 [Reference]	NA	1 [Reference]	NA
Tertile 2 (0.169-0.221)	32	35	1.83 (0.82-4.07)	.14	1.78 (0.80-3.99)	.16	1.57 (0.63-3.92)	.33
Tertile 3 (0.222-0.557)	33	42	2.15 (1.00-4.65)	.05	2.08 (0.95-4.53)	.07	3.37 (1.30-8.72)	.01
*P* for trend^b^	NA	NA	NA	.06	NA	.07	NA	.01
Quartile analysis^a^								
Quartile 1 (0.116-0.149)	24	11	1 [Reference]	NA	1.00 [Reference]	NA	1 [Reference]	NA
Quartile 2 (0.150-0.182)	23	14	1.22 (0.44-3.42)	.7	1.24 (0.43-3.40)	.69	1.72 (0.49-6.06)	.40
Quartile 3 (0.183-0.234)	24	36	3.59 (1.39-9.24)	.008	3.58 (1.39-9.24)	.008	3.89 (1.29-11.76)	.02
Quartile 4 (0.235-0.557)	24	34	3.47 (1.32-9.10)	.01	3.42 (1.30-8.99)	.01	8.37 (2.41-29.03)	.001
*P* for trend^b^	NA	NA	NA	.003	NA	.004	NA	<.001

Abbreviations: BMI, body mass index (calculated as weight in kilograms divided by height in meters squared); mtDNA, mitochondrial DNA; mtDNAfb, mitochondrial DNA fraction with breaks; NA, not applicable; OR, odds ratio.

^a^ All categories calculated based on distribution among controls.

^b^ *P* for trend calculated through analysis of indicator variables to test for ordinal trend.

We replicated results from Lan et al^7^ with regard to mtDNAcn with a dose-dependent increased risk of NHL in those in the highest tertile of mtDNAcn (eTable 5 in Supplement 1). Notably, further adjusting the mtDNAfb models for mtDNAcn yielded similar results (median OR, 4.03; 95% CI, 1.74-9.33), tertiles (*P* = .01 for trend), and quartiles (*P* < .001 for trend) (Table 2). There was a suggestion of an interaction, albeit not statistically significant, between mtDNAcn and mtDNAfb; however, the precision was limited with a small sample size in each category (eTable 6 in Supplement 1).

## Discussion

Our study is the first, to our knowledge, to demonstrate that increased mtDNAfbs are associated with a dose-response increased risk of developing NHL. This increased future risk was observed for mtDNAfb tertiles and quartiles, furthering the support of this dose-response association. Our study expands on prior findings that mtDNAcn is associated with the risk of NHL,^7^ while suggesting that mtDNAfb may also represent a biomarker of genomic instability or risk for oxidative stress as well as an etiologic risk factor for NHL.

Our study expands on previous research by using a population of men who smoke to determine whether mtDNAfb, indicative of oxidative stress and damage to the mitochondria, may be a biomarker for future risk of NHL.^12^ Fragmentation of mtDNA occurs after environmental stress and leads to deletions within the mitochondrial genome and loss of mtDNA content.^9,12^ Loss of mtDNA has been implicated in breast cancer^19^ but has not yet been evaluated in relation to NHL. This study suggests that an increased number of mtDNAfbs is associated with an increased future risk of NHL. Previous studies^12,20,21^ have recognized that mtDNAfb may be associated with loss of mtDNA content. With the loss of mtDNA content linked to multiple cancer types, our finding of increased mtDNAfb with increased risk of NHL supports the notion that loss of mtDNA content is implicated in NHL as well. Latency periods for development of NHL after exposure to environmental carcinogens are not well understood, with our study demonstrating an increased risk of NHL with increased mtDNAfb even after controlling for latent disease at entry into the ATBC Study, suggesting a potential role of mtDNAfb as an indicator of early disease.^22^

Diagnosis of NHL at this time is reliant on a lymph node biopsy with peripheral flow cytometry analyzing surface markers on B cells.^23^ This biopsy involves a small surgical procedure and specialized laboratory staff trained to perform flow cytometry. Both mtDNAfb and mtDNAcn have the potential to contribute to risk prediction models and stratification analyses to identify those at high risk for disease before biopsy. The US Preventive Services Task Force currently has no published guidelines on screening for NHL. Improved understanding of biomarkers placing patients at risk for NHL will provide health practitioners with the ability to better determine the need for such invasive procedures required for diagnosis of NHL with no known screening regimen.

### Strengths and Limitations 

This study has several strengths. Our study design—a prospective, nested case-control study using prediagnostic specimens—strengthens the interpretation of our results. A particular strength of the study is the analysis of prediagnostic peripheral white blood cell DNA, which reduces the possibility that latent disease might have altered mtDNAfb. Furthermore, the prospective design of this study also minimizes recall bias of questionnaire-based reporting, as well as biases related to sample collection, given that all questionnaire data and biological samples were collected at the beginning of the study for the entire cohort before NHL diagnosis.

This study also has some limitations. Our ability to conduct NHL subtype-specific analyses was hindered by both our sample size and the proportion of NHL (not otherwise specified) diagnoses. With more lymphoma cases currently being classified, future analyses may be able to identify greater numbers of NHL to allow researchers to conduct NHL subtype–specific analyses.^24,25,26^ Our nested case-control study was robustly designed to test the a priori hypothesis that mtDNAfb was associated with risk of NHL. This design, however, may hinder direct evaluation of the influence of specific confounders, such as age. This study was able to evaluate the risk of NHL and mtDNAfb while removing likely confounders, such as age and smoking, through matching. Because of matching, we are unable to evaluate the association between risk of NHL and the matching factors. Understanding the association of age with mtDNAfb and NHL risk would be of interest in future studies. Similarly, changing smoking habits and the impact of passive smoking habits on the risk of NHL could not be evaluated in this study; further study is needed to understand these possible associations in NHL.^27,28^ Furthermore, our study is limited in terms of generalizability given that it included only male Finnish smokers. Additional prospective studies are needed to evaluate the association between mtDNAfb and risk of NHL in a larger, more inclusive, ethnically diverse population.

## Conclusions

In summary, to the best of our knowledge, we are first to report that increased mtDNAfb was associated with increased future risk of NHL in a dose-dependent manner, indicating that mtDNAfb may be a useful biomarker for future risk of this hematologic cancer. Additional studies are needed to replicate our findings, especially in more diverse populations, including women and nonsmokers.

## Notes

Supplement 1. **eTable 1.** Correlation of Mitochondrial DNA Fractions With Breaks (mtDNAfb) With Mitochondrial DNA (mtDNA) Copy Number, Body Mass Index, Number of Cigarettes per Day, and Total Pack Years (Rho) **eTable 2.** Risk of Non-Hodgkin Lymphoma (NHL) Associated With Mitochondrial DNA Fraction With Breaks (mtDNAfb) Adjusted for Alpha-Tocopherol, Beta-Carotene Cancer Prevention Study (ATBC) Treatment Arm **eTable 3.** Risk of Non-Hodgkin Lymphoma (NHL) Associated With Mitochondrial DNA Fraction With Breaks (mtDNAfb) Excluding Cases (and Matched Controls) Who Were Diagnosed With NHL ≤365 Days From Blood Draw **eTable 4.** Risk of Non-Hodgkin Lymphoma (NHL) Associated With Mitochondrial DNA Fraction With Breaks (mtDNAfb) Excluding Cases (and Matched Controls) Who Were Diagnosed With NHL ≤730 Days From Blood Draw **eTable 5.** Risk of Non-Hodgkin Lymphoma (NHL) Based on Mitochondrial DNA (mtDNA) Copy Number **eTable 6.** Risk of Non-Hodgkin Lymphoma (NHL) With Mitochondrial DNA Fraction With Breaks (mtDNAfb) Stratified by Mitochondrial DNA (mtDNA) Copy Number

Supplement 2.
Data Sharing Statement
