# Supplementary material for: Mitochondrial DNA Fragmentation and Risk of Non-Hodgkin Lymphoma
Source: JAMA Netw Open. 2023 Aug 2;6(8):e2326885. doi: 10.1001/jamanetworkopen.2023.26885 (PMC10398405; doi:10.1001/jamanetworkopen.2023.26885)
Supplement: Supplement 1. — eTable 1. Correlation of Mitochondrial DNA Fractions With Breaks (mtDNAfb) With Mitochondrial DNA (mtDNA) Copy Number, Body Mass Index, Number of Cigarettes per Day, and Total Pack Years (Rho) eTable 2. Risk of Non-Hodgkin Lymphoma (NHL) Associated With Mitochondrial DNA Fraction With Breaks (mtDNAfb) Adjusted for Alpha-Tocopherol, Beta-Carotene Cancer Prevention Study (ATBC) Treatment Arm eTable 3. Risk of Non-Hodgkin Lymphoma (NHL) Associated With Mitochondrial DNA Fraction With Breaks (mtDNAfb) Excluding Cases (and Matched Controls) Who Were Diagnosed With NHL ≤365 Days From Blood Draw eTable 4. Risk of Non-Hodgkin Lymphoma (NHL) Associated With Mitochondrial DNA Fraction With Breaks (mtDNAfb) Excluding Cases (and Matched Controls) Who Were Diagnosed With NHL ≤730 Days From Blood Draw eTable 5. Risk of Non-Hodgkin Lymphoma (NHL) Based on Mitochondrial DNA (mtDNA) Copy Number eTable 6. Risk of Non-Hodgkin Lymphoma (NHL) With Mitochondrial DNA Fraction With Breaks (mtDNAfb) Stratified by Mitochondrial DNA (mtDNA) Copy Number [file jamanetwopen-e2326885-s001.pdf]

Supplementary Online Content

Hosgood HD III, Davitt M, Cawthon R, et al. Mitochondrial DNA fragmentation and risk of non-Hodgkin lymphoma. *JAMA Netw Open*. 2023;6(8):e2326885. doi:10.1001/jamanetworkopen.2023.26885

eTable 1. Correlation of Mitochondrial DNA Fractions With Breaks (mtDNAfb) With Mitochondrial DNA (mtDNA) Copy Number, Body Mass Index, Number of Cigarettes per Day, and Total Pack Years (Rho)
eTable 2. Risk of Non-Hodgkin Lymphoma (NHL) Associated With Mitochondrial DNA Fraction With Breaks (mtDNAfb) Adjusted for Alpha-Tocopherol, Beta-Carotene Cancer Prevention Study (ATBC) Treatment Arm
eTable 3. Risk of Non-Hodgkin Lymphoma (NHL) Associated With Mitochondrial DNA Fraction With Breaks (mtDNAfb) Excluding Cases (and Matched Controls) Who Were Diagnosed With NHL ≤365 Days From Blood Draw
eTable 4. Risk of Non-Hodgkin Lymphoma (NHL) Associated With Mitochondrial DNA Fraction With Breaks (mtDNAfb) Excluding Cases (and Matched Controls) Who Were Diagnosed With NHL ≤730 Days From Blood Draw
eTable 5. Risk of Non-Hodgkin Lymphoma (NHL) Based on Mitochondrial DNA (mtDNA) Copy Number
eTable 6. Risk of Non-Hodgkin Lymphoma (NHL) With Mitochondrial DNA Fraction With Breaks (mtDNAfb) Stratified by Mitochondrial DNA (mtDNA) Copy Number

This supplementary material has been provided by the authors to give readers additional information about their work.

© 2023 Hosgood HD III et al. *JAMA Network Open.*

eTable 1. Correlation of Mitochondrial DNA Fractions With Breaks (mtDNAfb) With Mitochondrial DNA (mtDNA) Copy Number, Body Mass Index, Number of Cigarettes per Day, and Total Pack Years (Rho)

**Correlation between mtDNAfb and selected variables**	**Controls**	***p*-value**	**Cases**	***p*-value**
Average mtDNA copy number	0.08	0.006	0.0025	0.63
Body Mass Index (kg/m^2)	0.13	0.22	0.15	0.14
Number of Cigarettes per day	0.02	0.81	0.10	0.31
Total Pack Years	0.05	0.63	0.09	0.37

© 2023 Hosgood HD III et al. *JAMA Network Open.*

eTable 2. Risk of Non-Hodgkin Lymphoma (NHL) Associated With Mitochondrial DNA Fraction With Breaks (mtDNAfb) Adjusted for Alpha-Tocopherol, Beta-Carotene Cancer Prevention Study (ATBC) Treatment Arm

**Average mitochondrial DNA fraction with breaks in placebo arm**	**Controls**	**Cases**	**OR[a] (95%CI)**
<median (0.116-0.2044)	12	5	1 (ref)
≥median (0.2045-0.557)	14	21	5.0 (0.58, 42.8)
Tertile 1 (0.118-0.168)	8	4	1 (ref)
Tertile 2 (0.169-0.221)	7	10	2.0 (0.18, 22.1)
Tertile 3 (0.222-0.545)	11	12	1.12×10^{8} (0, inf)
p_{trend}			0.15
**Average mitochondrial DNA fraction with breaks in alpha-tocopherol treatment arm**	**Controls**	**Cases**	**OR[a] (95%CI)[a]**
<median (0.116-0.2044)	11	6	1 (ref)
≥median (0.2045-0.557)	11	19	2.5×10^{17} (0, inf)
Tertile 1 (0.118-0.168)	8	4	1 (ref)
Tertile 2 (0.169-0.221)	7	11	did not converge
Tertile 3 (0.222-0.545)	7	10	did not converge
p_{trend}			0.57
**Average mitochondrial DNA fraction with breaks in beta-carotene arm**	**Controls**	**Cases**	**OR[a] (95%CI)[a]**
<median (0.116-0.2044)	14	9	1 (ref)
≥median (0.2045-0.557)	11	13	3 (0.31, 28.8)
Tertile 1 (0.118-0.168)	7	5	1 (ref)
Tertile 2 (0.169-0.221)	10	11	2.31 (0.16, 33.2)
Tertile 3 (0.222-0.545)	8	6	1.52 (0.066, 34.8)
p_{trend}			0.71
**Average mitochondrial DNA fraction with breaks in alpha-tocopherol and beta-carotene treatment arm**	**Controls**	**Cases**	**OR[a] (95%CI)[a]**
<median (0.116-0.2044)	10	5	1 (ref)
≥median (0.2045-0.557)	12	17	did not converge
Tertile 1 (0.118-0.168)	7	5	1 (ref)
Tertile 2 (0.169-0.221)	8	3	did not converge
Tertile 3 (0.222-0.545)	7	14	did not converge
p_{trend}			did not converge

[a]ORs and 95% Cis determined by conditional logistic regression

© 2023 Hosgood HD III et al. *JAMA Network Open.*

eTable 3. Risk of Non-Hodgkin Lymphoma (NHL) Associated With Mitochondrial DNA Fraction With Breaks (mtDNAfb) Excluding Cases (and Matched Controls) Who Were Diagnosed With NHL ≤365 Days From Blood Draw

mtDNAfb[d]	Controls	Cases	OR(95%CI)[a]	OR(95%CI)[b]	OR(95%CI)[c]
<median (0.116-0.2044)	47	22	1.00 (ref)	1.00 (ref)	1.00 (ref)
≥median (0.2045-0.557)	48	60	3.63 (1.66, 7.93)	3.57 (1.61, 7.92)	4.13 (1.68, 10.13)
Tertile 1 (0.118-0.168)	30	16	1.00 (ref)	1.00 (ref)	1.00 (ref)
Tertile 2 (0.169-0.221)	32	30	1.98 (0.83, 4.72)	1.93 (0.80, 4.64)	1.68 (0.65, 4.35)
Tertile 3 (0.222-0.557)	33	36	2.39 (1.04, 5.51)	2.32 (0.99, 5.43)	3.01 (1.14, 7.96)
p_{trend}			0.04	0.06	0.03
Quartile analysis					
Quartile 1 (0.116-0.14)	24	9	1.00 (ref)	1.00 (ref)	1.00 (ref)
Quartile 2 (0.15-0.182)	23	13	1.56 (0.50, 4.85)	1.64 (0.51, 5.27)	1.78 (0.47, 6.68)
Quartile 3 (0.183-0.234)	24	31	4.18 (1.49, 11.76)	4.16 (1.46, 11.82)	4.16 (1.29, 13.41)
Quartile 4 (0.235-0.557)	24	29	4.98 (0.65, 15.00)	5.11 (1.66, 15.72)	9.14 (2.39, 34.98)
p_{trend}			0.001	0.002	0.001

[a]Odds Ratio (OR) and 95% Confidence Intervals (CI) determined by conditional logistic regression

[b]ORs and 95% CIs determined by conditional logistic regression adjusted for pack years of smoking, number of cigarettes per day and BMI

[c]ORs and 95% CIs determined by conditional logistic regression adjusted for mtDNA copy number, pack years of smoking, number of cigarettes per day and BMI

[d]Analysis excluded those diagnosed within first year of blood draw

© 2023 Hosgood HD III et al. *JAMA Network Open.*

eTable 4. Risk of Non-Hodgkin Lymphoma (NHL) Associated With Mitochondrial DNA Fraction With Breaks (mtDNAfb) Excluding Cases (and Matched Controls) Who Were Diagnosed With NHL ≤730 Days From Blood Draw

mtDNAfb[d]	Controls	Cases	OR(95%CI)[a]	OR(95%CI)[b]	OR(95%CI)[c]
<median (0.116-0.2044)	47	21	1.00 (ref)	1.00 (ref)	1.00 (ref)
≥median (0.2045-0.557)	47	47	2.63 (1.16, 5.93)	2.57 (1.13, 5.87)	2.84 (1.14, 7.05)
Tertile analysis					
Tertile 1 (0.118-0.168)	30	15	1.00 (ref)	1.00 (ref)	1.00 (ref)
Tertile 2 (0.169-0.221)	32	26	1.54 (0.61, 3.85)	1.46 (0.58, 3.70)	1.15 (0.42, 3.12)
Tertile 3 (0.222-0.557)	33	27	1.66 (0.68, 4.03)	1.58 (0.64, 3.89)	1.87 (0.68, 5.13)
p_{trend}			0.27	0.33	0.22
Quartile analysis					
Quartile 1 (0.116-0.14)	24	9	1.00 (ref)	1.00 (ref)	1.00 (ref)
Quartile 2 (0.15-0.182)	23	12	1.27 (0.38, 4.23)	1.22 (0.36, 4.18)	1.29 (0.34, 4.88)
Quartile 3 (0.183-0.234)	24	25	2.66 (0.91, 7.77)	2.52 (0.86, 7.43)	2.41 (0.72, 8.06)
Quartile 4 (0.235-0.557)	24	22	3.48 (1.09, 11.14)	3.38 (1.05, 10.88)	5.88 (1.45, 23.88)
p_{trend}			0.02	0.02	0.009

[a]Odds Ratio (OR) and 95% Confidence Intervals (CI) determined by conditional logistic regression

[b]ORs and 95% CIs determined by conditional logistic regression adjusted for pack years of smoking, number of cigarettes per day and BMI

[c]ORs and 95% CIs determined by conditional logistic regression adjusted for mtDNA copy number, pack years of smoking, number of cigarettes per day and BMI

[d]Analysis excluded those diagnosed within first 730 days of blood draw

© 2023 Hosgood HD III et al. *JAMA Network Open.*

eTable 5. Risk of Non-Hodgkin Lymphoma (NHL) Based on Mitochondrial DNA (mtDNA) Copy Number

**mtDNA copy number**	**Controls**	**Cases**	**OR(95%CI)[a]**	**OR(95%CI)[b]**	**OR(95%CI)[c]**
<median (0.624-0.943)	49	30	1.00 (ref)	1.00 (ref)	1.00 (ref)
≥median (0.944-2.78)	48	67	2.73 (1.37, 5.44)	4.03 (1.81, 8.96)	2.96 (1.45, 6.07)
Tertile 1 (0.651-0.883)	33	25	1.00 (ref)	1.00 (ref)	1.00 (ref)
Tertile 2 (0.884-0.96)	32	20	0.85 (0.39, 1.87)	0.88 (0.40, 1.97)	0.98 (0.41, 2.35)
Tertile 3 (0.97-1.71)	32	52	2.46 (1.15, 5.30)	2.75 (1.23, 6.14)	3.90 (1.61, 9.43)
p_{trend}			0.015	0.01	0.002
Quartile analysis					
Quartile 1 (0.116-0.14)	25	21	1.00 (ref)	1.00 (ref)	1.00 (ref)
Quartile 2 (0.15-0.182)	24	9	0.44 (0.16, 1.23)	0.58 (0.21, 1.62)	0.48 (0.16, 1.47)
Quartile 3 (0.183-0.234)	23	20	1.24 (0.50, 3.04)	1.33 (0.53, 3.33)	1.81 (0.67, 4.90)
Quartile 4 (0.235-0.557)	25	47	2.97 (1.20, 7.30)	3.36 (1.30, 8.69)	5.16 (1.81, 14.73)
p_{trend}			0.005	0.003	0.001

[a]Odds Ratio (OR) and 95% Confidence Intervals (CI) determined by conditional logistic regression
[b]Odds Ratio and 95% Confidence Intervals determined by conditional logistic regression adjusted for smoking status and BMI
[c]ORs and 95% CIs determined by conditional logistic regression adjusted for average mtDNA fraction with breaks, pack years of smoking

© 2023 Hosgood HD III et al. *JAMA Network Open.*

eTable 6. Risk of Non-Hodgkin Lymphoma (NHL) With Mitochondrial DNA Fraction With Breaks (mtDNAfb) Stratified by Mitochondrial DNA (mtDNA) Copy Number

**Median mtDNAfb**[a]	**Low mtDNA copy number**[b]		**High median mtDNA copy number**[b]		
	Cases/controls	OR[c] (95% CI)[c]	Cases/Controls	OR[c](95% CI)[c]	*P*-interaction
Low	7/18	1.00 (ref)	18/29	2.41 (0.75, 7.80)	
High	22/29	2.31 (0.71, 7.50)	48/19	21.14 (0.40, 1119)	0.11

[a]Based on median distribution of mtDNA fraction with breaks in controls ((0.116-0.2044 vs 0.2045-0.557)

[b]Based on median distribution of mtDNA copy number in controls (0.624-0.943 vs 0.944-2.78)
[c]Odds Ratio (OR) and 95% Confidence Intervals (CI) determined by conditional logistic regression adjusted for number of cigarettes per day, pack years of smoking, and BMI

© 2023 Hosgood HD III et al. *JAMA Network Open.*
