# Supplementary material for: Mitochondrial DNA Fragmentation and Risk of Non-Hodgkin Lymphoma
Source: JAMA Netw Open. 2023 Aug 2;6(8):e2326885. doi: 10.1001/jamanetworkopen.2023.26885 (PMC10398405; doi:10.1001/jamanetworkopen.2023.26885)
Supplement: Supplement 2. — Data Sharing Statement [file jamanetwopen-e2326885-s002.pdf]

Data Sharing Statement

Hosgood. Mitochondrial DNA Fragmentation and Risk of Non-Hodgkin Lymphoma. *JAMA Netw Open*. Published August 02, 2023. doi:10.1001/jamanetworkopen.2023.26885

Data
Data available: Yes
Data types: Data dictionary, Deidentified participant data, Data (not involving human participants)
How to access data: dean.hosgood@einsteinmed.edu
When available: With publication

Supporting Documents
Document types: Statistical/analytic code
How to access documents: dean.hosgood@einsteinmed.edu
When available: With publication

Additional Information
Who can access the data: Upon reasonable request
Types of analyses: Upon reasonable purpose
Mechanisms of data availability: with investigator support
